# Two new xanthones and cytotoxicity from the bark of *Garcinia schomburgkiana*

**DOI:** 10.1007/s11418-018-1240-8

**Published:** 2018-08-31

**Authors:** Sutin Kaennakam, Kamonwan Mudsing, Kitiya Rassamee, Pongpun Siripong, Santi Tip-pyang

**Affiliations:** 1 Center of Excellence in Natural Products Chemistry, Department of Chemistry, Faculty of Science, Chulalongkorn University, Bangkok, 10330 Thailand; 2 Natural Products Research Section, Research Division, National Cancer Institute, Bangkok, 10400 Thailand

**Keywords:** *Garcinia schomburgkiana*, Clusiaceae, Xanthone, Cytotoxicity

## Abstract

Two new xanthone derivatives, named schomburgones A (**1**) and B (**2**), along with eight known compounds, including xanthones (**3**–**8**) and anthraquinones (**9**–**10**) were isolated from the bark of *Garcinia schomburgkiana*. Their structures were determined by spectroscopic analysis especially 1D and 2D NMR spectroscopies. All isolated compounds were evaluated for their cytotoxicity against five cancer cell lines (KB, HeLa S-3, HT-29, MCF-7 and HepG-2). Compounds **3**–**6** and **8** showed good cytotoxicity against all the five cancer cell lines with IC_50_ values in the range of 1.45–9.46 µM.

**Electronic supplementary material:**

The online version of this article (10.1007/s11418-018-1240-8) contains supplementary material, which is available to authorized users.

## Introduction

*Garcinia schomburgkiana* Pierre (family Clusiaceae) is a medium-sized tree distributed in Thailand, Laos, Vietnam, and Cambodia. In folk medicine in these countries, its leaves, roots, and fruits are used for the treatment of cough, menstrual disturbances, expectorant, laxative and diabetes [1]. Previous chemical and biological studies on the chemical constituents of *G. schomburgkiana* showed the presence of xanthones, depsidones, biphenyls, flavonoids, triterpenoids, and phloroglucinols, some of which exhibited antimalarial activity and cytotoxicity [2], [3]. Here, we reported two new xanthone derivatives, named schomburgones A (**1**) and B (**2**), along with six known xanthones (**3**–**8**) and two known anthraquinones (**9**–**10**) from the bark of this plant. The structures of all isolated compounds were elucidated using spectroscopic methods especially 1D and 2D NMR spectroscopies and compared with their ^1^H and ^13^C NMR spectroscopic data from the literature. The cytotoxicity of all isolated compounds was evaluated using the MTT method against five cancer cell lines.

## Results and discussion

**Fig. 1:**
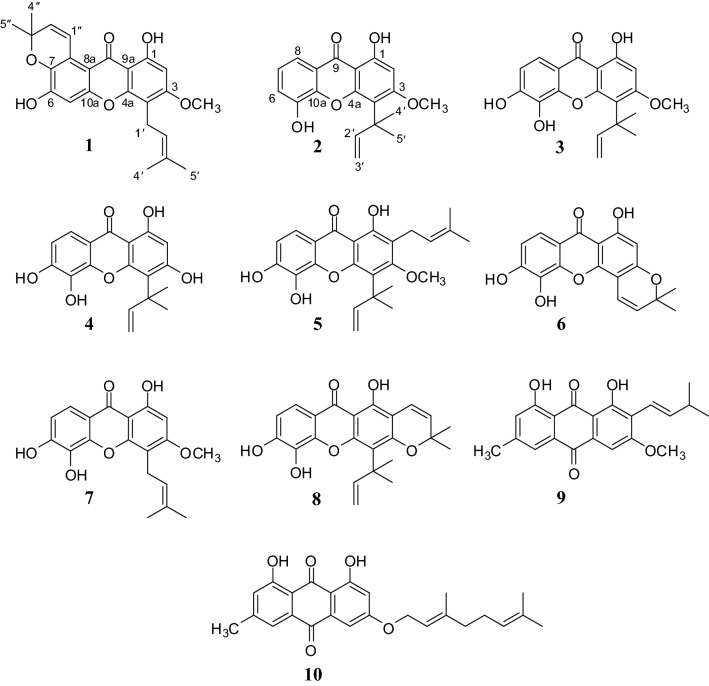
Chemical structures of **1**–**10**

Phytochemical investigation of CH_2_Cl_2_ crude extract from the bark of *G. schomburgkiana* led to the isolation of two new xanthone derivatives, named schomburgones A (**1**) and B (**2**), along with eight known compounds (Fig. 1), including isocudraniaxanthone B (**3**) [4], gerontoxanthone I (**4**) [5], nigrolineaxanthone E (**5**) [6], isojacareubin (**6**) [7], dulxanthone A (**7**) [8], macluraxanthone (**8**) [9], vismiaquinone A (**9**) [10], and 3-geranylemodin (**10**) [11]. The structures of all isolated compounds were elucidated using spectroscopic methods especially NMR spectroscopies and compared with their ^1^H and ^13^C NMR spectroscopic data from the literature.

**Fig. 2:**
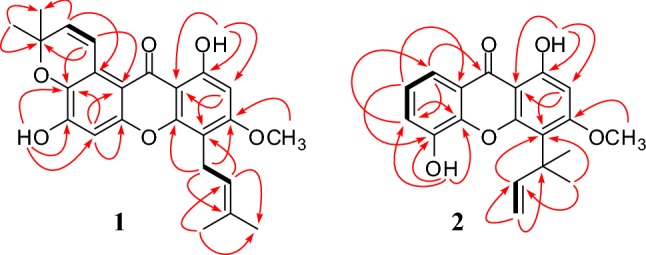
Key HMBC (arrow curves) and COSY (bold lines) correlations of **1** and **2**

Schomburgone A (**1**) was obtained as yellow oil. Its molecular formula was determined as C_24_H_24_O_6_ by the negative HRESIMS measurement through the ion peak at *m/z* 407.1527 [M–H]^+^ (calcd. for C_24_H_23_O_6_, 407.1495). The UV spectrum displayed absorption bands at *λ*_max_ 395, 315 and 243 nm, which is typical of the xanthone chromophore [12]. The IR spectrum showed phenolic hydroxyl groups and a hydrogen bonded carbonyl group at 3422 and 1632 cm^−1^. The ^1^H NMR spectrum showed the presence of a 3,3-dimethylallyl substituent, which was confirmed by two singlets at *δ*_H_ 1.67 (3H, s, H-4*′*) and 1.85 (3H, s, H-5*′*) for the vinyl methyls, a triplet at *δ*_H_ 5.20 (1H, t, *J *= 7.23 Hz, H-2*′*) for the vinylic proton and a doublet at *δ*_H_ 3.44 (2H, d, *J *= 7.23 Hz, H-1*′*) for the allylic proton of prenyl group. In addition, the methoxy signal, a hydroxyl signal, two aromatic proton signals and a hydrogen bonded hydroxyl signal appeared as five singlets at *δ*_H_ 3.89 (3H, s, OCH_3_-3), 6.26 (1H, s, OH-6), 6.33 (1H, s, H-2), 6.85 (1H, s, H-5) and 13.38 (1H, s, OH-1), respectively. The signals at *δ*_H_ 1.50 (6H, s, H-4*″* and H-5*″*), 5.83 (1H, d, *J *= 10.23 Hz, H-2*″*) and 8.02 (1H, d, *J *= 10.23 Hz, H-1*″*) in the spectrum were indicative of a dimethylchromene ring. The angular fusion of the chromene ring at C-8 was deduced from the low field shift of H-1*″* (*δ*_H_ 8.02), which was located in the deshielding area of the carbonyl group. The ^1^H and ^13^C NMR spectroscopic data (Table 1) were shown to be similar to those of the known xanthone, paxanthone B [13], except that the hydroxyl group at C-3 of paxanthone B was replaced by a methoxy group. In the HMBC correlations of **1** (Fig. 2), the methoxy proton at *δ*_H_ 3.89 showed a cross-peak with *δ*_C_ 163.5 (C-3). In addition, a methine proton at *δ*_H_ 8.02 showed cross-peaks with *δ*_C_ 77.1 (C-3*″*), 108.5 (C-8a), and 136.9 (C-7), confirming that a dimethylchromene ring was located at C-8, and a methylene proton at *δ*_H_ 3.44 showed cross-peaks with *δ*_C_ 131.7 (C-3*′*), 153.7 (C-4a), and 163.5 (C-3), indicating that a prenyl group was attached to C-4. Thus, the complete assignment of schomburgone A was determined as **1**.

**Table 1:** NMR spectroscopic data (400 MHz, CDCl_3_) for **1** and **2**

Position	**1**		**2**	
	*δ*_H_ (*J* in Hz)	*δ*_C_	*δ*_H_ (*J* in Hz)	*δ*_C_
1		162.0		163.0
2	6.33 (s)	94.1	6.43 (s)	96.2
3		163.5		166.1
4		107.2		113.9
4a		153.7		154.0
5	6.85 (s)	102.6		145.9
6		151.1	7.26 (d, 7.62)	120.3
7		136.9	7.23 (t, 7.62)	124.6
8		119.9	7.71 (d, 7.62)	116.6
8a		108.5		121.0
9		183.0		182.0
9a		103.9		104.1
10a		153.5		144.8
1*′*	3.44 (d, 7.23)	21.7		42.0
2*′*	5.20 (t, 7.23)	122.4	6.70 (dd, 10.63,17.67)	156.3
3*′*		131.7	5.22 (d, 17.67), 5.07 (d, 10.63)	104.5
4*′*	1.67 (s)	25.9	1.61 (s)	28.4
5*′*	1.85 (s)	18.0	1.61 (s)	28.4
1*″*	8.02 (d, 10.23)	121.2		
2*″*	5.83 (d, 10.23)	132.5		
3*″*		77.1		
4*″*	1.50 (s)	27.5		
5*″*	1.50 (s)	27.5		
1-OH	13.38 (s)		13.25 (s)	
5-OH			6.42 (s)	
6-OH	6.26 (s)			
3-OCH_3_	3.89 (s)	56.1	3.91 (s)	56.2

Schomburgone B (**2**) was obtained as yellow oil. Its molecular formula was determined as C_19_H_18_O_5_ by the negative HRESIMS measurement through the ion peak at *m/z* 325.1098 [M–H]^+^ (calcd. for C_19_H_17_O_5_, 325.1076). The UV spectrum displayed absorption bands at *λ*_max_ 394, 315 and 244 nm. The IR spectrum showed phenolic hydroxyl groups and a hydrogen bonded carbonyl group at 3432 and 1642 cm^−1^. The ^1^H NMR spectrum showed the presence of a 1,1-dimethylallyl group, which was confirmed by a singlets at *δ*_H_ 1.61 (6H, s, H-4*′* and H-5*′*) for two methyls, a doublet of doublet at *δ*_H_ 6.70 (1H, dd, *J *= 10.63 Hz, 17.67, H-2*′*) for the methine proton and two doublets at *δ*_H_ 5.07 (1H, d, *J *= 10.63 Hz, H-3*′*) and 5.22 (1H, d, *J *= 17.67 Hz, H-3*′*) for the methylene protons. Moreover, the methoxy signal, hydroxyl signal, aromatic proton signal and hydrogen bonded hydroxyl signal appeared as four singlets at *δ*_H_ 3.91 (3H, s, OCH_3_-3), 6.42 (1H, s, OH-5), 6.43 (1H, s, H-2) and 13.25 (1H, s, OH-1), respectively. The ABC-type aromatic protons were assigned at *δ*_H_ 7.23 (1H, t, *J *= 7.62 Hz, H-7), 7.26 (1H, d, *J *= 7.62 Hz, H-6) and 7.71 (1H, d, *J *= 7.62 Hz, H-8). The ^1^H and ^13^C NMR spectroscopic data (Table 1) were shown to be similar to those of the known xanthone, pancixanthone A [14], except that the hydroxyl group at C-3 of pancixanthone-A was substituted by a methoxy group. In the HMBC correlations of **2** (Fig. 2), the methoxy proton at *δ*_H_ 3.91 showed a cross-peak with *δ*_C_ 166.1 (C-3). Moreover, two methyl protons at *δ*_H_ 1.61 showed cross-peaks with *δ*_C_ 113.9 (C-4) and 156.3 (C-2*′*), confirming that a 1,1-dimethylallyl group was connected at C-4. Thus, the completed assignment of schomburgone B was determined as **2**.

In previous research many xanthones showed cytotoxicity [15]. Therefore, all isolated compounds were evaluated in vitro for their cytotoxicity against five cancer cell lines (KB, HeLa S-3, HT-29, MCF-7 and HepG-2) (Table 2). Compounds **3**–**6** and **8** showed good cytotoxicity against all five cancer cell lines with IC_50_ values in the range of 1.45–9.46 µM. Compounds **1** and **7** showed weak cytotoxicity against all five cancer cell lines with IC_50_ values in the range of 34.69–73.10 µM. Compounds **2**, **9** and **10** showed inactive cytotoxicity against all five cancer cell lines with IC_50_ values >100 µM. The SAR studied data (Fig. 1; Table 2) of xanthones suggest that the ortho hydroxy group at C-5 and C-6 and the 1,1-dimethylallyl group at C-4 might improve the cytotoxicity as inferred from the comparison of their cytotoxicity of compounds **1**–**10**.

**Table 2:** In vitro cytotoxicity of compounds **1**–**10** against five cancer cell lines

Compounds	IC_50_ (μM) ± SD				
	KB	HeLa S-3	HT-29	MCF-7	HepG-2
**1**	45.05 ± 2.08	69.22 ± 4.02	61.92 ± 2.40	52.21 ± 1.71	73.19 ± 1.14
**2**	> 100	> 100	> 100	> 100	> 100
**3**	5.23 ± 0.19	7.95 ± 0.25	7.87 ± 0.30	6.70 ± 0.81	5.93 ± 0.94
**4**	4.69 ± 0.21	7.57 ± 0.26	9.18 ± 0.38	5.26 ± 0.55	4.89 ± 0.83
**5**	5.08 ± 0.36	5.82 ± 0.15	4.17 ± 0.07	7.19 ± 0.36	9.46 ± 0.45
**6**	4.30 ± 0.12	6.60 ± 0.24	5.92 ± 0.40	3.21 ± 0.71	3.19 ± 0.14
**7**	38.17 ± 6.83	65.26 ± 3.89	34.69 ± 2.29	46.03 ± 1.29	54.80 ± 1.18
**8**	1.45 ± 0.09	1.62 ± 0.20	1.87 ± 0.30	1.70 ± 0.81	1.93 ± 0.94
**9**	> 100	> 100	> 100	> 100	> 100
**10**	> 100	> 100	> 100	> 100	> 100
Doxorubicin	0.13 ± 0.006	0.03 ± 0.001	0.31 ± 0.07	0.42 ± 0.14	1.23 ± 0.02

IC_50_ ≤ 10 = good activity, 10 < IC_50_ ≤ 30 = moderate activity, IC_50_ > 100 = inactive

## Experimental

### General experimental procedures

NMR spectra were recorded on Bruker 400 AVANCE spectrometer. HRESIMS spectra were obtained using a Bruker MICROTOF model mass spectrometer. The UV–visible absorption spectra were recorded on a UV-2550 UV–Vis spectrometer (Shimadzu, Kyoto, Japan). The IR spectra were measured on a Nicolet 6700 FT-IR spectrometer using KBr discs.

### Plant material

The bark of *G. schomburgkiana* was collected from Bang Ramat Road, Khwaeng Bang Ramat, Khet Taling Chan, Bangkok Thailand (13°45*′*42*″*N, 100°24*′*56*″*E), in June 2017. The plant material was identified by Dr. Suttira Sedlak, a botanist at the Walai Rukhavej Botanical Research Institute, Mahasarakham University, and a specimen retained as a reference (Khumkratok no. 92-08).

### Extraction and isolation

The air-dried bark of *G. schomburgkiana* (2.0 kg) was extracted with CH_2_Cl_2_ at room temperature for 7 days (2 × 25 L). The CH_2_Cl_2_ crude extract (91.0 g) was further separated by column chromatography (CC) over silica gel CC and eluted with a gradient of Hexane–EtOAc (90, 70, 50 and 30% Hexane–EtOAc each 5 L) to give six fractions (A-F). Fraction A (4.0 g) was purified by Sephadex LH-20 column eluted with 80% CH_2_Cl_2_–MeOH (2 L) and further applied to a radial chromatography (chromatotron) with 95% hexane–EtOAc (200 mL) to afford compound **9** (3.2 mg). Fraction B (10.5 g) was purified by Sephadex LH-20 column eluted with 80% CH_2_Cl_2_–MeOH (2 L) and further applied to a chromatotron with 50% hexane–CH_2_Cl_2_ (200 mL) to obtain compounds **2** (4.2 mg), **4** (2.5 mg) and **7** (2.3 mg). Compound **1** (7.2 mg) was separated by Sephadex LH-20 column eluted with 50% CH_2_Cl_2_–MeOH (2 L) from fraction C (2.0 g). Fraction D (6.5 g) was purified by Sephadex LH-20 column eluted with 50% CH_2_Cl_2_–MeOH (2 L) to give compounds **5** (8.5 mg) and **8** (4.6 mg). Fraction E (8.5 g) was purified by Sephadex LH-20 column eluted with 50% CH_2_Cl_2_–MeOH (2 L) and further applied to a chromatotron with 70% hexane–EtOAc (200 mL) to obtain compounds **3** (5.2 mg) and **10** (5.5 mg). Finally, fraction F (1.2 g) was subjected to silica gel CC eluted with 100% CH_2_Cl_2_ and further purified by Sephadex LH-20 column eluted with 80% CH_2_Cl_2_–MeOH (2 L) to yield compound **6** (6.5 mg).

*Schomburgone A (1)*: yellow oil; UV (CHCl_3_) *λ*_max_ (log ε): 395 (3.6), 315 (4.2) and 243 (4.4) nm,. IR ν_max_ (KBr): 3422 and 1632 cm^−1^; ^1^H (400 MHz, CDCl_3_) and ^13^C NMR (100 MHz, CDCl_3_) spectroscopic data, see Table 1; HRESIMS *m/z* 407.1527 [M–H]^+^ (calcd. for C_24_H_23_O_6_, 407.1495).

*Schomburgone B (2)*: yellow oil; UV (CHCl_3_) *λ*_max_ (log ε): 394 (3.5), 315 (4.0) and 244 (4.2) nm,. IR ν_max_ (KBr): 3432 and 1642 cm^−1^; ^1^H (400 MHz, CDCl_3_) and ^13^C NMR (100 MHz, CDCl_3_) spectroscopic data, see Table 1; HRESIMS *m/z* 325.1098 [M–H]^+^ (calcd. for C_19_H_17_O_5_, 325.1076).

### Cytotoxicity assay

All isolated compounds (**1**–**10**) were subjected to cytotoxic evaluation against KB (human epidermoid carcinoma), HeLa S-3 (human cervical carcinoma), HT-29 (human colon adenocarcinoma), MCF-7 (human breast adenocarcinoma) and HepG-2 (human liver carcinoma) cell lines employing the colorimetric method [16]. Doxorubicin was used as the reference substance which exhibits activity against five cancer cell lines. The 3-(4,5-dimethylthiazol-2-yl)-2,5-diphenyl-tetrazolium bromide (Sigma Chemical Co., USA) was dissolved in saline to make a 5 mg/mL stock solution. Cancer cells (3 × 10^3^ cells) suspended in 100 μg/wells of MEM medium containing 10% fetal calf serum (Gibco BRL, Life Technologies, NY, USA) were seeded onto a 96-well culture plate (Costar, Corning Incorporated, NY, USA). After 24 h pre-incubation at 37 °C in a humidified atmosphere of 5% CO_2_/95% air to allow cellular attachment, various concentrations of test solution (0.1, 0.3, 1.0, 3.0, 10.0, 30.0, and 100.0 μM, each 10 μL/well) were added and these were then incubated for 48 h under the above conditions. At the end of the incubation, 10 μL of tetrazolium reagent was added into each well followed by further incubation at 37 °C for 4 h. The supernatant was decanted, and DMSO (100 μL/well) was added to allow formosan solubilization. The optical density of each well was detected using a Microplate reader at 550 nm and for correction at 595 nm. Each determination represented the average mean of six replicates. The 50% inhibition concentration (IC_50_ value) was determined by curve fitting.

## Electronic supplementary material

Below is the link to the electronic supplementary material.

Supplementary material 1 (PDF 972 kb)
