# Supplementary material for: Two new xanthones and cytotoxicity from the bark of Garcinia schomburgkiana
Source: J Nat Med. 2018 Aug 31;73(1):257–61. doi: 10.1007/s11418-018-1240-8 (PMC6326088; doi:10.1007/s11418-018-1240-8)
Supplement: Supplementary file 1 — Supplementary material 1 (PDF 972 kb) [file 11418_2018_1240_MOESM1_ESM.pdf]

Supplementary Material

Two new xanthones and cytotoxicity from the bark of *Garcinia schomburgkiana*

Sutin Kaennakam [1,*]. Kamonwan Mudsing [1]. Kitiya Rassamee [2]. Pongpun Siripong [2]. Santi Tip-pyang [1,*]

[1]*Center of Excellence in Natural Products Chemistry, Department of Chemistry, Faculty of Science, Chulalongkorn University, Bangkok 10330, Thailand*
[2]*Natural Products Research Section, Research Division, National Cancer Institute, Bangkok 10400, Thailand*

Corresponding authors

Assoc. Prof. Dr. Santi Tip-pyang, Tel.: +66 89 7917596; E-mail address: santi.Ti@chula.ac.th
Dr.Sutin Kaennakam, Tel.; +66 91 4142346; E-mail address: n-s-k-@hotmail.com

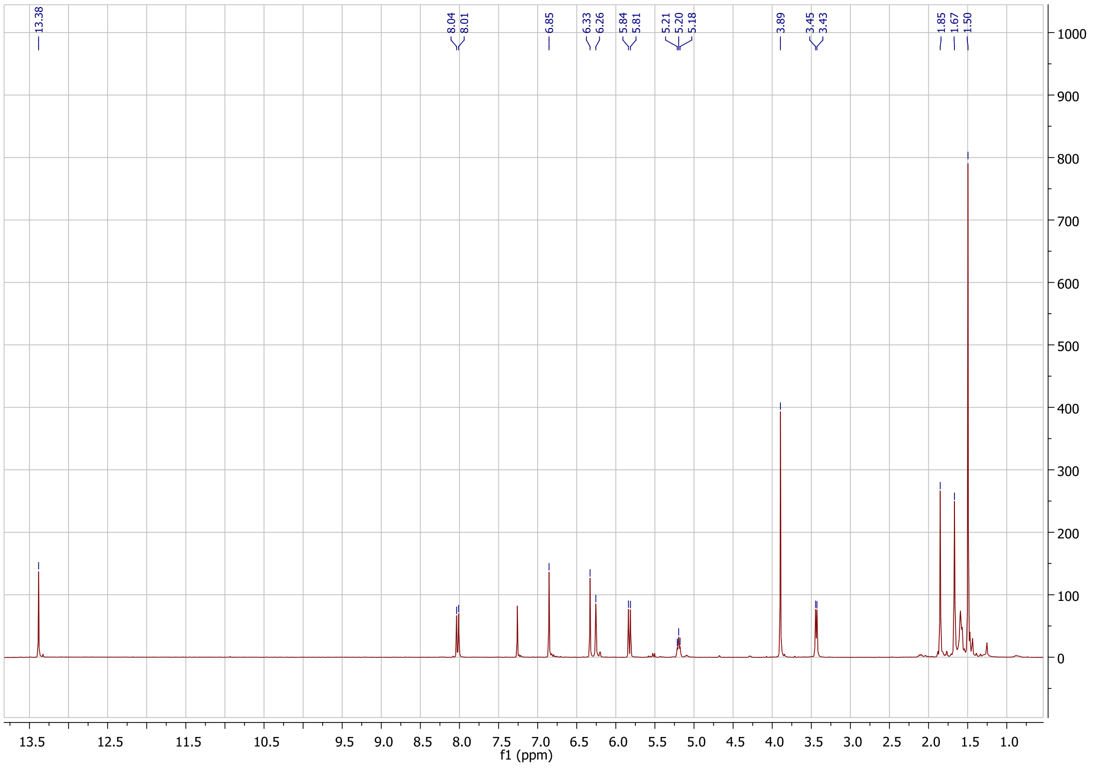

Figure S.1 ^{1}H NMR spectrum of **1** in $CDCl_3$

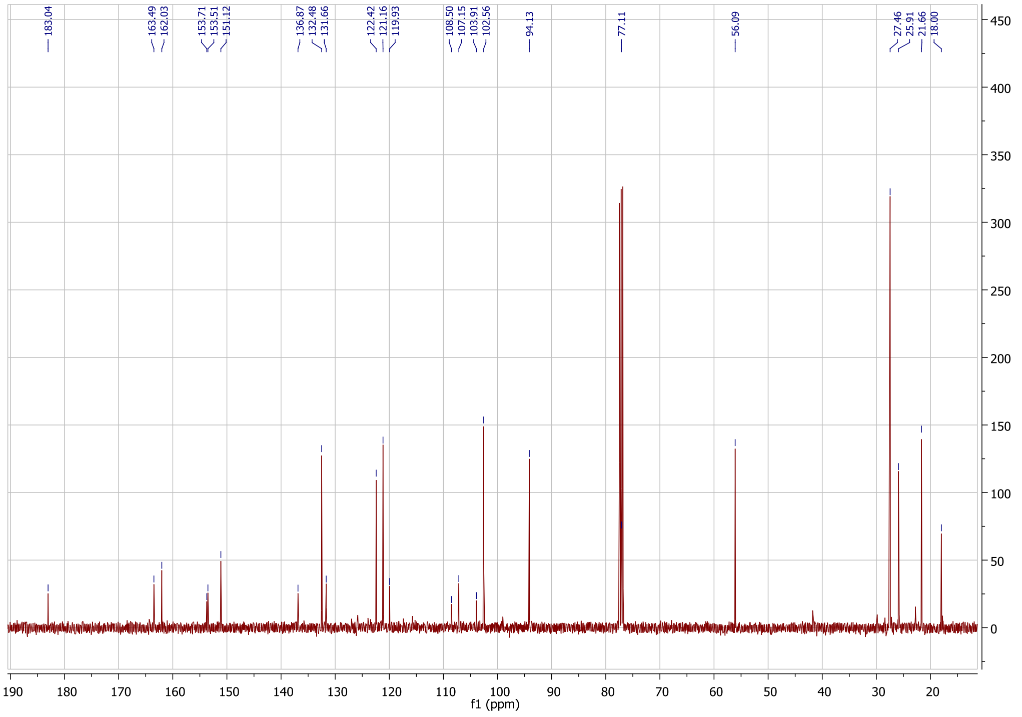

Figure S.2 ^{13}C NMR spectrum of **1** in $CDCl_3$

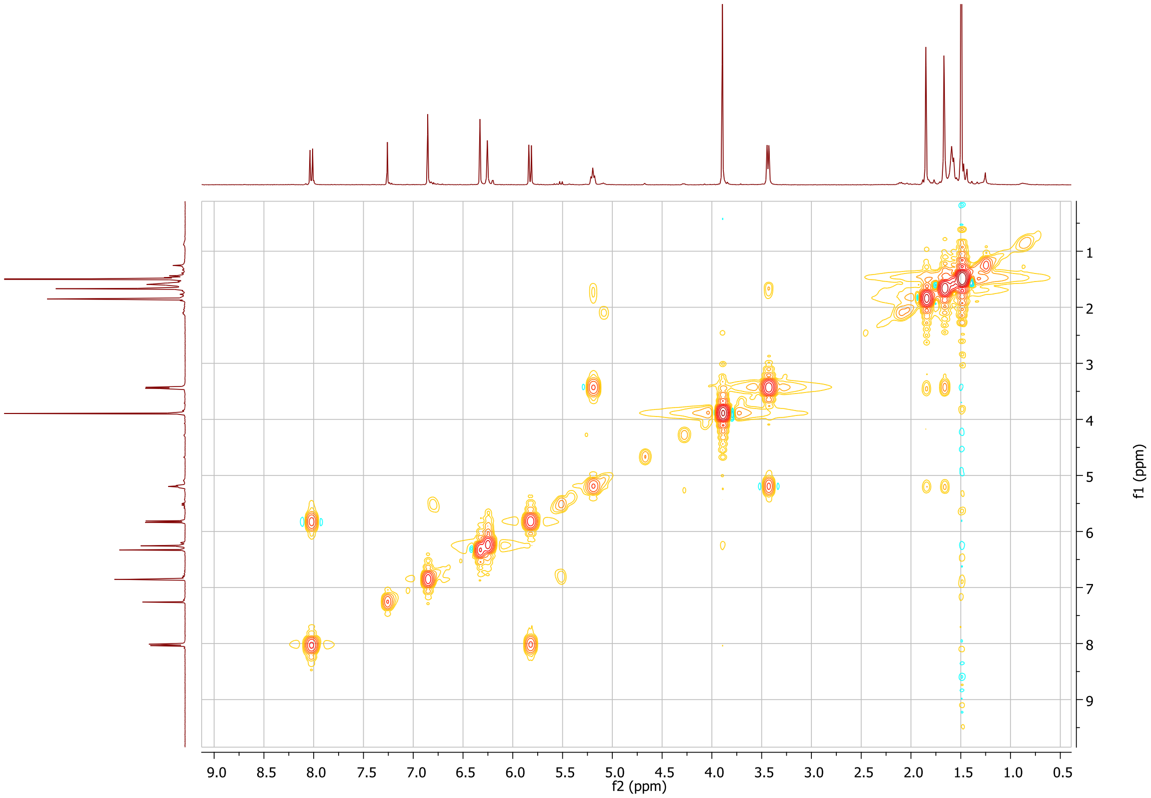

Figure S.3 COSY NMR spectrum of **1** in $CDCl_3$

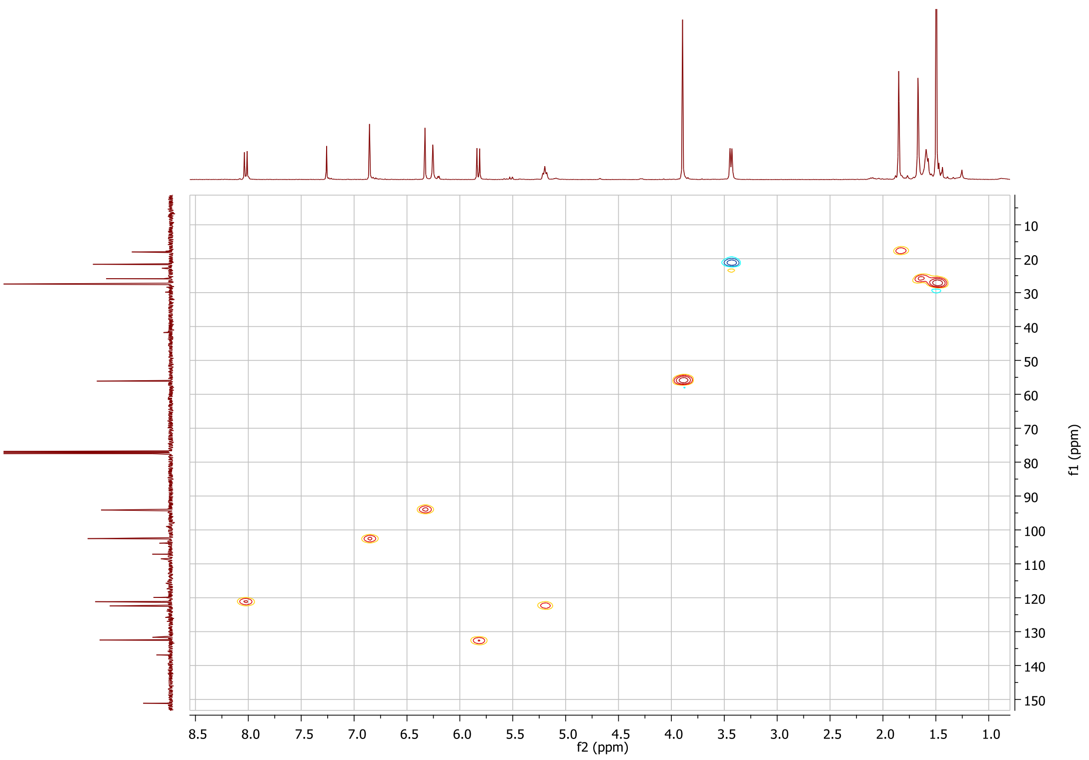

Figure S.4 HSQC NMR spectrum of **1** in $CDCl_3$

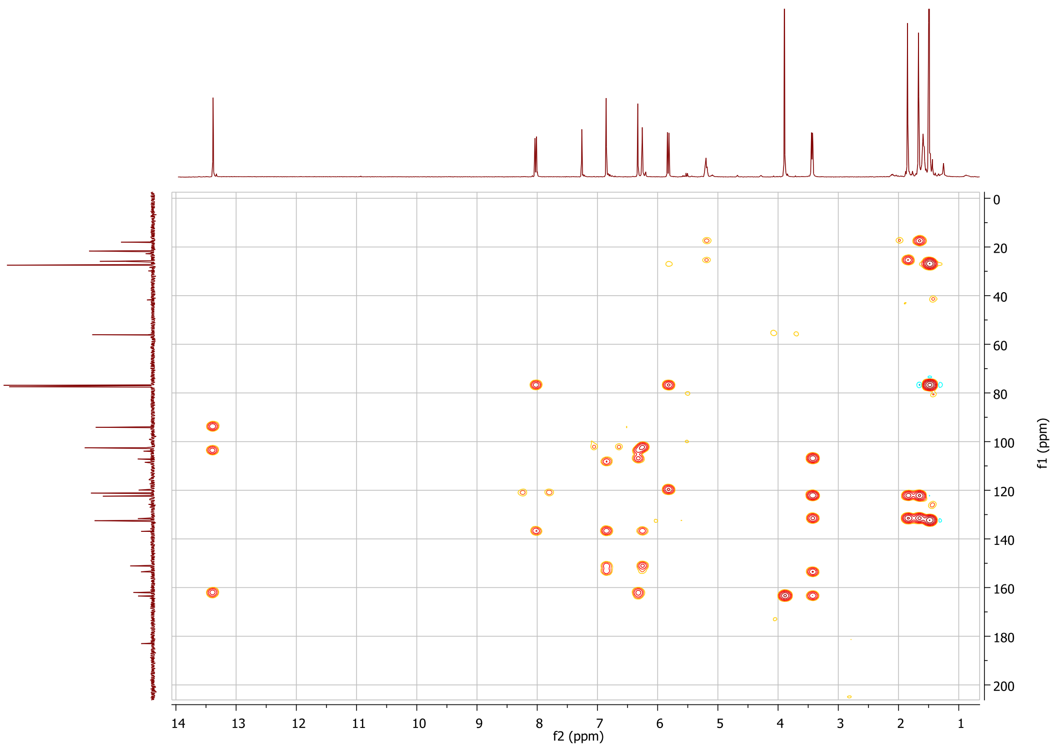

Figure S.5 HMBC NMR spectrum of **1** in $CDCl_3$

Mass Spectrum List Report

Analysis Info

Analysis Name	D:\Data\Data Service\180330_neg_GST1.d	Acquisition Date	3/30/2018 2:49:14 PM
Method	NV_neg_0.3min_profile_1segment_lowNubulizerDrygas(2).m	Operator	CU.
Sample Name	180330_neg_GST1	Instrument / Ser#	micrOTOF-Q II 10335
Comment			

Acquisition Parameter

Source Type	ESI	Ion Polarity	Negative	Set Nebulizer	0.4 Bar
Focus	Not active	Set Capillary	4000 V	Set Dry Heater	200 ℃
Scan Begin	50 m/z	Set End Plate Offset	-500 V	Set Dry Gas	4.0 l/min
Scan End	1500 m/z	Set Collision Cell RF	150.0 Vpp	Set Divert Valve	Waste

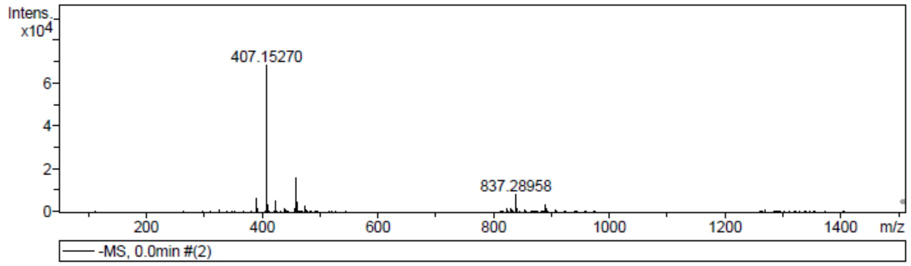

#	m/z	Res.	S/N	I	FWHM
1	325.11046	8740	56.1	617	0.03720
2	391.11966	8787	587.2	6459	0.04451
3	392.12466	8698	155.1	1706	0.04508
4	407.15270	8194	6222.5	68448	0.04969
5	408.15461	9193	1676.9	18446	0.04440
6	409.15659	8716	297.6	3274	0.04694
7	423.14904	8822	451.1	4962	0.04797
8	424.15208	8647	107.6	1184	0.04905
9	439.14125	9456	109.9	1209	0.04644
10	441.15279	8747	90.6	997	0.05043

Figure S.6 Mass spectrum of **1**

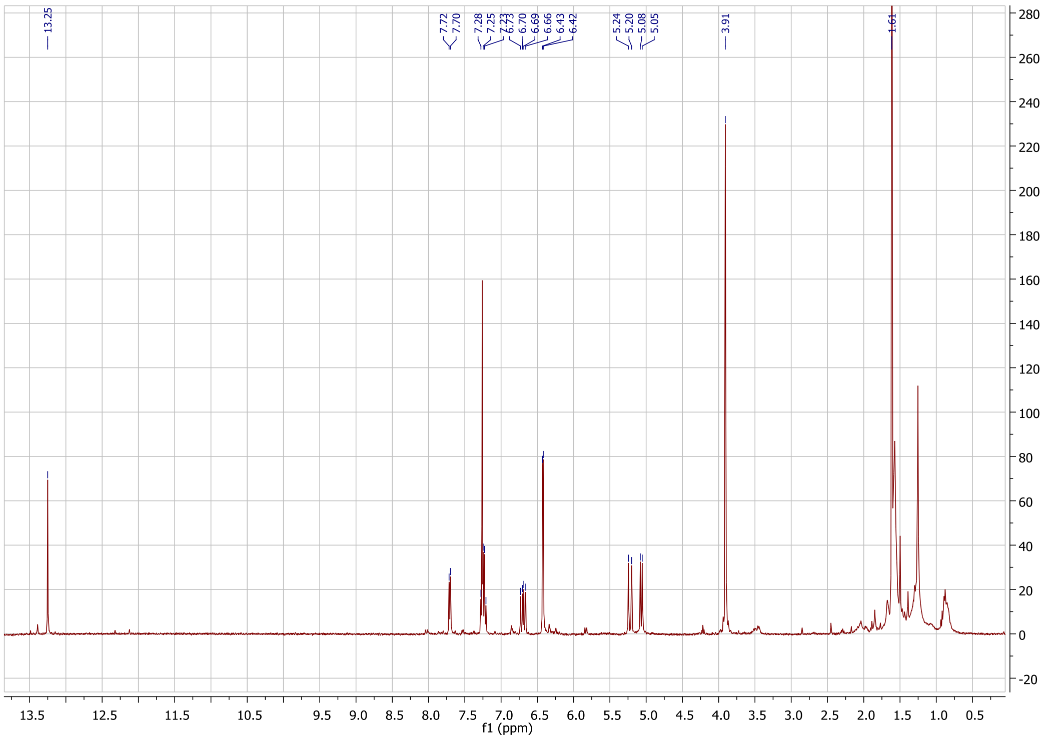

Figure S.7 ^{1}H NMR spectrum of **2** in $CDCl_3$

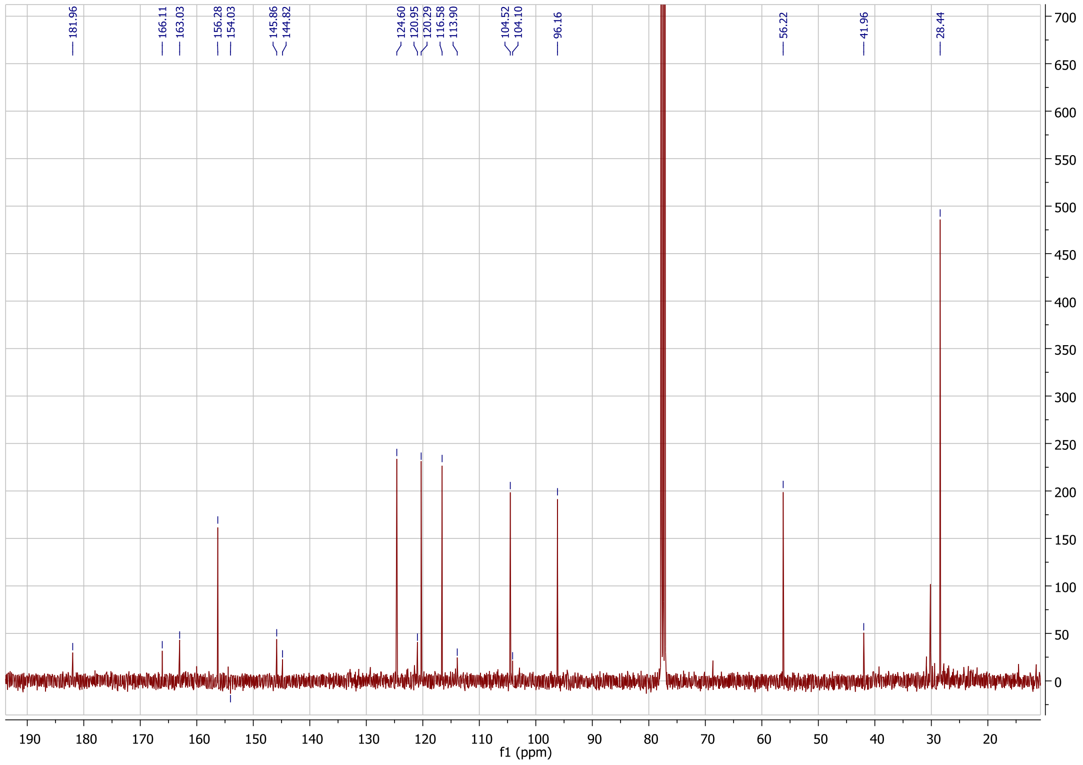

Figure S.8 ^{13}C NMR spectrum of **2** in $CDCl_3$

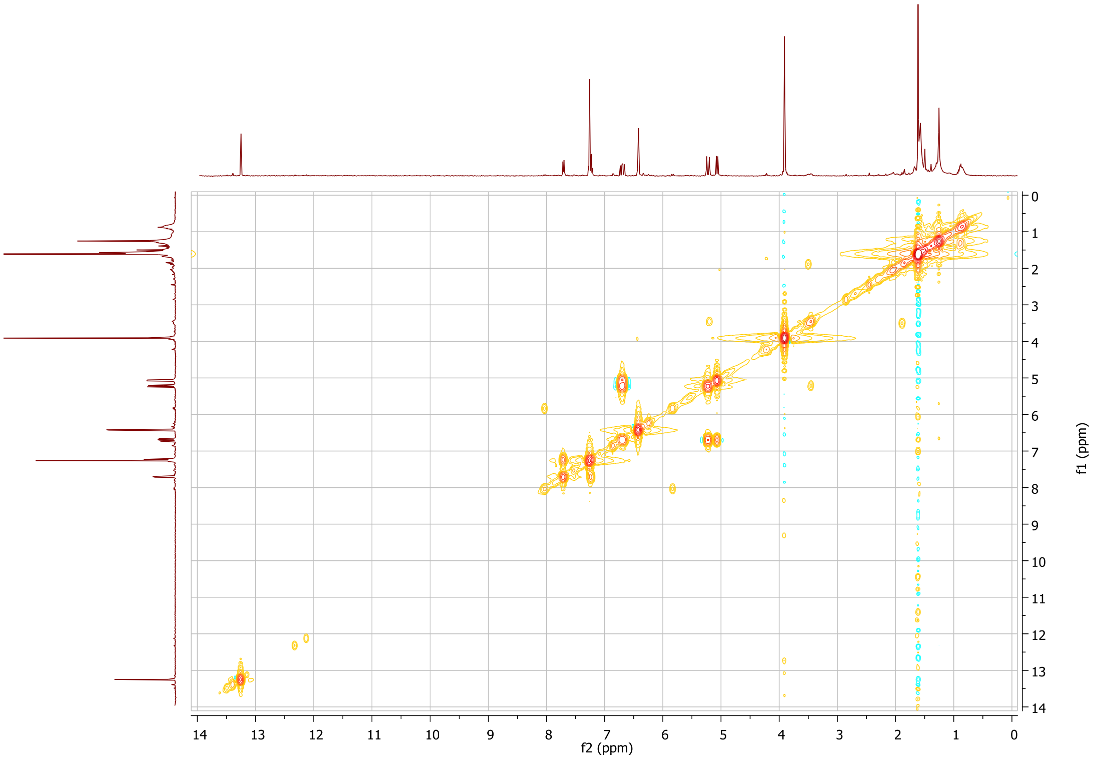

Figure S.9 COSY NMR spectrum of **2** in $CDCl_3$

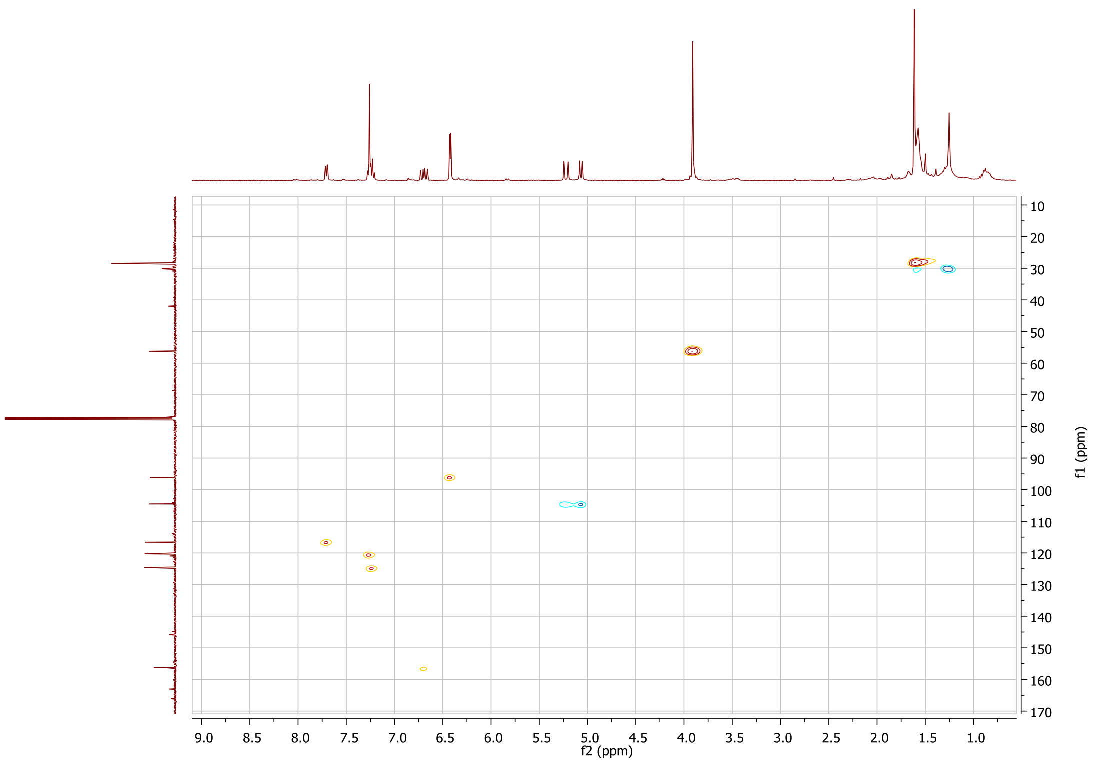

Figure S.10 HSQC NMR spectrum of **2** in $CDCl_3$

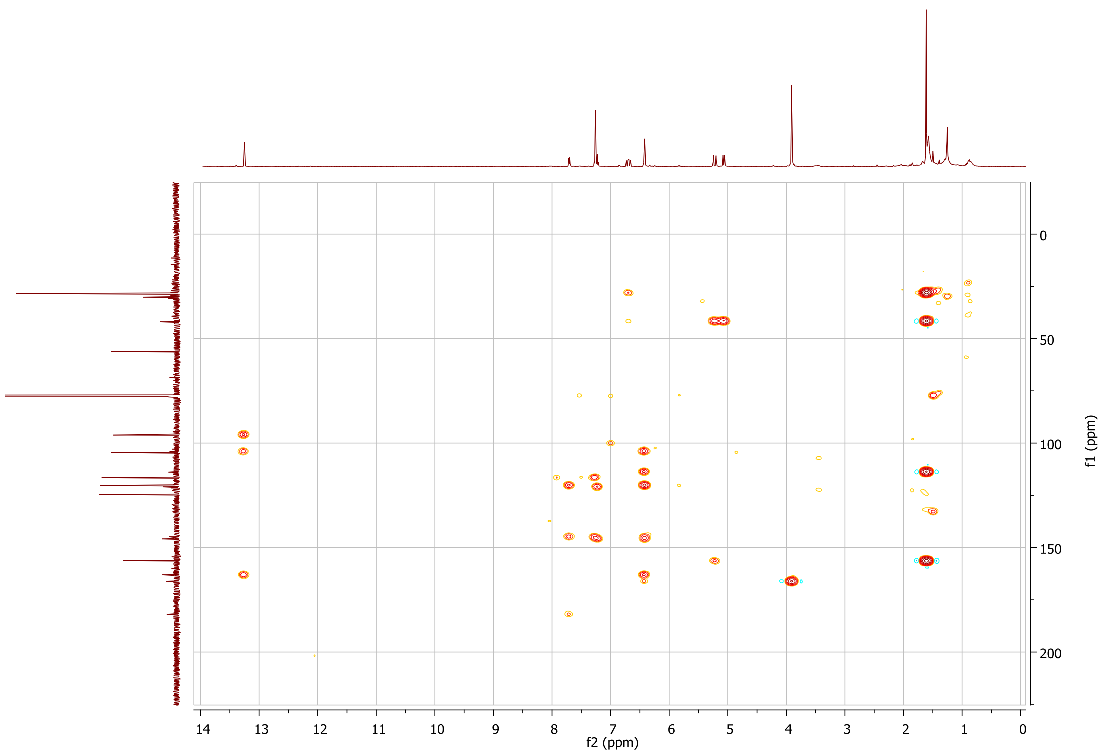

Figure S.11 HMBC NMR spectrum of **2** in $CDCl_3$

Mass Spectrum List Report

Analysis Info

Analysis Name	D:\Data\Data Service\180330_neg_GST2.d	Acquisition Date	3/30/2018 3:32:30 PM
Method	NV_neg_0.3min_profile_1segment_lowNubulizerDrygas(2).m	Operator	CU.
Sample Name	180330_neg_GST2	Instrument / Ser#	micrOTOF-Q II 10335
Comment			

Acquisition Parameter

Source Type	ESI	Ion Polarity	Negative	Set Nebulizer	0.4 Bar
Focus	Not active	Set Capillary	4000 V	Set Dry Heater	200 ℃
Scan Begin	50 m/z	Set End Plate Offset	-500 V	Set Dry Gas	4.0 l/min
Scan End	1500 m/z	Set Collision Cell RF	150.0 Vpp	Set Divert Valve	Waste

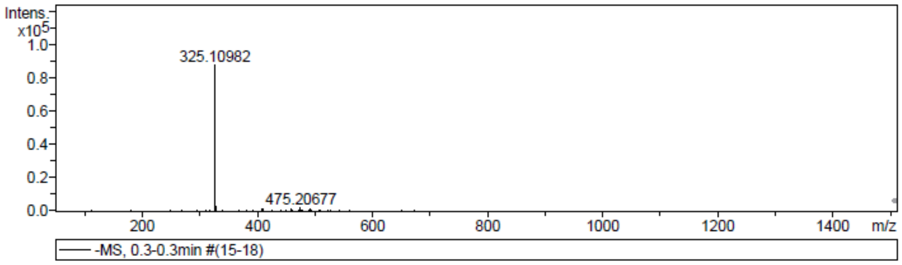

#	m/z	Res.	S/N	I	FWHM
1	112.98520	5634	181.1	498	0.02005
2	248.95932	7848	82.1	226	0.03172
3	269.04641	7606	87.8	242	0.03538
4	295.06007	8551	103.1	284	0.03451
5	310.08310	8393	81.7	225	0.03695
6	311.09293	8656	196.8	541	0.03594
7	325.10982	7604	31758.2	87335	0.04276
8	326.11206	8649	6810.3	18728	0.03770
9	327.11352	8547	981.1	2698	0.03827
10	328.11542	7977	110.6	304	0.04113

Figure S.12 Mass spectrum of **2**
